# Estimability before accuracy: missing-data strategies for classroom-scale student assessment

**DOI:** 10.3389/fpsyg.2026.1902834

**Published:** 2026-09-30

**Authors:** Atilla Özdemir

**Affiliations:** Department of Mathematics and Science Education, Faculty of Education, Süleyman Demirel University, Isparta, Türkiye

**Keywords:** classroom assessment, estimability, item response theory, item response tree, missing data, small samples

## Abstract

Guidance on handling missing item responses in educational assessments is drawn almost entirely from large-scale testing programs; however, much of the practical work occurs in single classes and small multi-class samples. This Monte Carlo study aimed to identify which strategies can be estimated at all at that scale, and how accurate they are once estimation succeeds. A complete-data benchmark and four missing-data strategies were evaluated across sample sizes from 40 to 200. The primary design examined four missingness mechanisms at a 15% missingness rate, and an additional 25% sensitivity condition was evaluated at selected sample sizes; each condition used 100 replications. The strategies were listwise deletion, a two-parameter logistic model fitted directly to the incomplete response matrix, multiple imputation followed by calibration, and a two-factor item response tree model. Estimability, not accuracy, separated the procedures. At 40 examinees, an admissible solution was obtained in 0.42 to 0.46 of replications with complete data, 0.19 to 0.23 for direct estimation, 0.02 to 0.07 for multiple imputation, and 0.00 to 0.05 for the item response tree model. The two-factor tree model showed severe estimation instability throughout the smallest sample-size region, and the same pattern persisted when data were generated from the tree model itself, ruling out misspecification as the sole explanation. Once estimation succeeded, the procedures differed by only a few hundredths of a theta standard deviation in root mean square error, while substantial individual-level uncertainty remained. Under the investigated conditions, direct estimation was more often estimable and showed a small accuracy advantage over the implemented imputation procedure in the adequately supported paired comparisons. At classroom scale, the choice of a missing-data strategy is primarily a question of estimability, and convergence and parameter stability should be reported whenever these models are applied.

## Introduction

Missing item responses are a routine obstacle when assessment data are used to evaluate students, programs, and instructional interventions. When learners omit items, the incomplete response matrix can distort the ability estimates on which evaluative decisions rest. The problem is especially consequential in classroom-scale evaluation, where applied samples can be far smaller than those examined in the item response tree (IRTree) simulation literature. At this scale, the analyst must choose a missing-data strategy with little guidance calibrated to the samples that are actually encountered because the methodological literature has developed around much larger assessments.

Four strategies are available in practice. Listwise deletion discards every examinee with any omission. It remains a software default but sacrifices cases and biases results unless data are missing completely at random (Enders, 2010; Little et al., 2014). A conventional item response theory model can be fitted directly to the incomplete matrix, using all observed responses and treating omissions as missing, without deleting examinees or imputing values. Pohl et al. (2014) discuss this approach, and Xiao and Bulut (2020) found it to perform best under most of the conditions they examined. Multiple imputation replaces missing values with draws from a conditional distribution and analyzes the completed data sets (Rubin, 1987; Schafer and Graham, 2002). Item response tree models treat an omission as informative rather than absent, decomposing the response into a participation node and a correctness node estimated jointly within item response theory (De Boeck and Partchev, 2012; Pohl et al., 2014) (see Figure 1).

**Figure 1:**
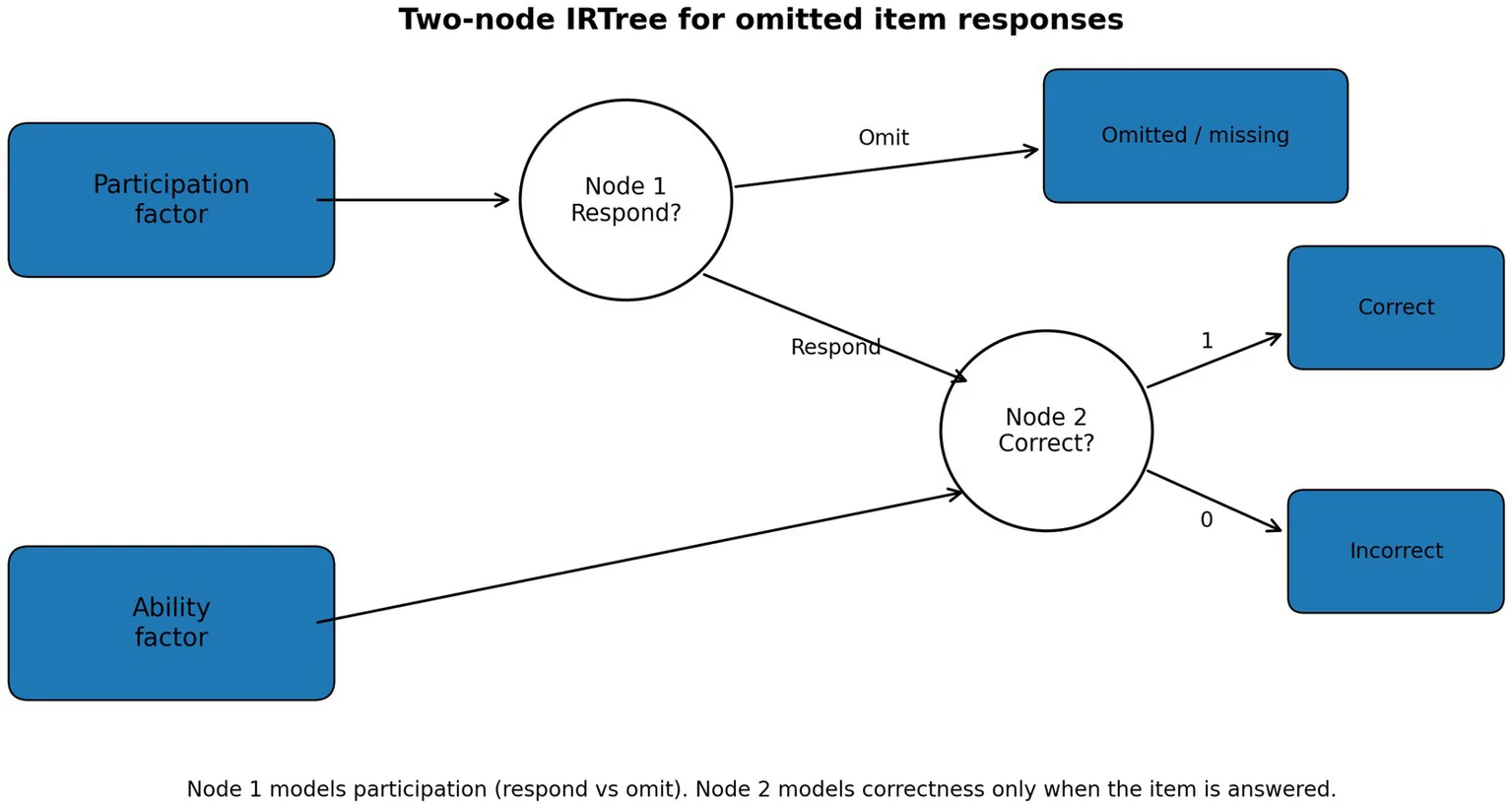
Two-node item response tree model for omitted item responses. The first node models the respond-or-omit decision and defines the participation factor. The second node models correctness and defines the ability factor.

Recent simulation evidence has favored the tree-based approach. Soğuksu and Demir (2025) compared it with the expectation–maximization algorithm and multiple imputation across samples of 400 to 1,500 and reported the lowest root mean square error for the tree model, concluding that sample size was not a substantial factor. Rose et al. (2017) and Debeer et al. (2017) reported reduced bias for tree-based treatments of omitted responses in large-scale settings. Whether any of these recommendations transfer to classroom-scale samples has not been examined. The smallest sample studied by Soğuksu and Demir (2025) was 400, an order of magnitude above most classroom studies, and studies that examine tree-model parameter recovery directly work at 500 examinees and above (Alarcon et al., 2023).

The present study examines that transfer. It differs from earlier comparisons in one respect that turns out to be decisive. Rather than asking only which procedure is most accurate, it asks first which procedures can be estimated at all and treats accuracy as a question that arises only once estimation has succeeded. Three questions were addressed. First, what is the probability of obtaining an admissible solution for each strategy across the classroom-scale range? Second, conditional on obtaining one, how well is student ability recovered, and how do the procedures compare? Third, where the two-factor tree model fails, and is the failure a property of the model at small samples or a consequence of fitting a model that does not correspond to the process generating the data?

### Theoretical background

#### Missing data mechanisms

Rubin (1976) distinguished three mechanisms. Missing completely at random occurs when missingness is unrelated to observed or unobserved values. Missing at random occurs when missingness depends on observed values only. Missing not at random occurs when missingness depends on the unobserved values themselves, the condition under which conventional treatments are most vulnerable (Little and Rubin, 2002). The distinction matters for simulation design as well as for analysis. Conditioning the missingness probability on the latent trait rather than on realized responses produces a mechanism that is more naturally regarded as non-ignorable, which is why Xiao and Bulut (2020) deliberately generated their missing-at-random condition from observed response information and Soğuksu and Demir (2025) used groups formed from observed total scores.

In the classroom assessment, non-ignorable missingness is plausible. Lower-achieving students may omit items they cannot answer, and timed tests produce ability-related nonresponse (Debeer et al., 2014). Alarcon et al. (2023) stressed the importance of evaluating missing-data methods under conditions that reflect realistic assessment settings.

#### Procedures for incomplete assessment data

Multiple imputation proceeds through imputation, analysis, and pooling (Rubin, 1987) and is implemented for chained equations in the mice package (Van Buuren and Groothuis-Oudshoorn, 2011). It is reliable under ignorable mechanisms (Graham, 2009; Schafer, 1997) but can be biased when the imputation model misrepresents the mechanism (Baraldi and Enders, 2010). An important feature of the procedure in an item response theory context is that imputation does not remove the need to calibrate a measurement model on the sample at hand. The completed data sets must still be calibrated, and that calibration inherits whatever difficulty the sample size imposes. Şahin and Anıl (2017) reported that, among the sample sizes they examined, 500 respondents were required for a 20-item two-parameter logistic test to meet their item-parameter recovery criteria. That figure is conditional on their data, estimation method, and criteria and is not a universal minimum, but it motivates explicit scrutiny of the calibration that underlies any imputation-based procedure at much smaller samples.

Item response tree models decompose each response into a sequence of binary decisions, each represented by a separate item response model (De Boeck and Partchev, 2012). For missing data, Pohl et al. (2014) modeled a respond-or-omit node followed by a correctness node, yielding a participation factor and an ability factor within a multidimensional framework (Reckase, 2009). Debeer et al. (2017) and Rose et al. (2017) found that the approach reduced bias under non-ignorable missingness; Jeon and De Boeck (2016) and Plieninger (2021) extended and operationalized it; and Alagöz and Meiser (2024) and Merhof and Meiser (2023) proposed mixture and dynamic extensions. Pohl and Becker (2020) showed that the performance of missing-data approaches depends strongly on the correspondence between the fitted model and the actual mechanism, which is directly relevant to any claim about why a tree model fails. The freely estimated two-factor specification must recover roughly 80 item parameters together with a factor correlation, and its behavior at classroom-scale samples has not been characterized.

## Materials and methods

### Analysis rules fixed in advance

Because this study evaluates estimation failure, the rules governing what counts as a successful estimate and how failures enter the comparison were written down and frozen before any results were produced. A timestamped record of the frozen protocol, together with a technical verification report, is provided in the Supplementary material.

A fit was recorded as admissible when three conditions held jointly: no fatal estimation error, a successful convergence indicator, and finite parameter estimates. Admissibility was an operational criterion for estimation completion and should not be interpreted as evidence of satisfactory parameter recovery or stability. A ceiling on item discrimination was deliberately not part of this definition, because using extreme discriminations to exclude solutions would make the central finding circular. Extreme discriminations were recorded separately as a stability diagnostic. All two-parameter logistic procedures used the same estimator and scoring method, with no fallback to an alternative estimator when a fit failed. If any item in an analyzed matrix had no observed responses or no observed variance, the replication was recorded as a failure rather than the item being dropped, and the replication remained in the denominator.

### Design

Item responses were generated from the two-parameter logistic model (Birnbaum, 1968) with 20 dichotomous items. Discriminations were drawn from a uniform distribution over 1 to 2, difficulties from a uniform distribution over −2 to 2, and abilities from a standard normal distribution, following the conventions used by Soğuksu and Demir (2025) and Demir and Parlak (2012). Sample sizes were 40, 60, 80, 100, 120, and 200. Four mechanisms were crossed with sample size at a 15% missingness rate, and a 25% condition was run at sample sizes of 40, 120, and 200. A further condition generated data from a two-factor tree model. The design comprised 39 conditions with 100 replications each. Each replication sets its own seed as a deterministic function of its condition index and replication number, so results do not depend on execution order or on the number of parallel workers.

### Missing data generation

Under the completely random mechanism, each cell was set to missing independently with probability equal to the target rate. The missing at random condition uses a response-score-based operationalization following Soğuksu and Demir (2025). The order of operations was as follows: complete responses were generated, a total score was computed from those complete responses, examinees were divided into three groups by that score, and group-specific missingness probabilities of 1.5, 1.0, and 0.5 times a common constant were assigned and rescaled so that the overall rate matched the target. The grouping variable is therefore a realized score rather than the latent parameter, which also respects the reasoning of Xiao and Bulut (2020). Under the response-based non-ignorable mechanism, the probability depended on the response itself: incorrect and correct responses were assigned relative missingness weights of 1.5 and 0.5, respectively, and the resulting probabilities were rescaled to match the target overall missingness rate. A fourth mechanism, in which the probability depended on the latent ability parameter, was retained as a separate non-ignorable sensitivity condition.

Quality control confirmed that the mechanisms behaved as intended. The realized missingness rate deviated from the target by at most 0.004 in every condition. Under the missing at random mechanism, the realized rates were approximately 0.21, 0.14, and 0.07 in the low, middle, and high groups. Under the response-based mechanism, omission was about three times more likely for incorrect than for correct responses. Under the latent sensitivity mechanism, the correlation between true ability and the individual missingness proportion was approximately negative 0.61.

### Procedures

Five procedures were evaluated. The complete-data benchmark fitted a two-parameter logistic model to the generated responses before missingness was imposed, within the same replication, so that it is paired with every other procedure rather than merely averaged alongside them. Listwise deletion was evaluated for feasibility and selectivity only; no model was fitted to the retained cases Figure 2. Direct estimation fitted a two-parameter logistic model to the incomplete matrix using all observed responses, without deleting examinees or imputing values, and it was verified at the code level that no examinee is removed. Multiple imputation used predictive mean matching with five imputations and ten iterations; each completed data set was calibrated separately, and person estimates were averaged across imputations. The interest here is recovery of the point estimate rather than inference about its variance, so imputation variance was not pooled. A replication counted as successful only if all five completed data sets yielded an admissible calibration, and every individual imputation was logged so that alternative rules could be evaluated without re-running the study.

**Figure 2:**
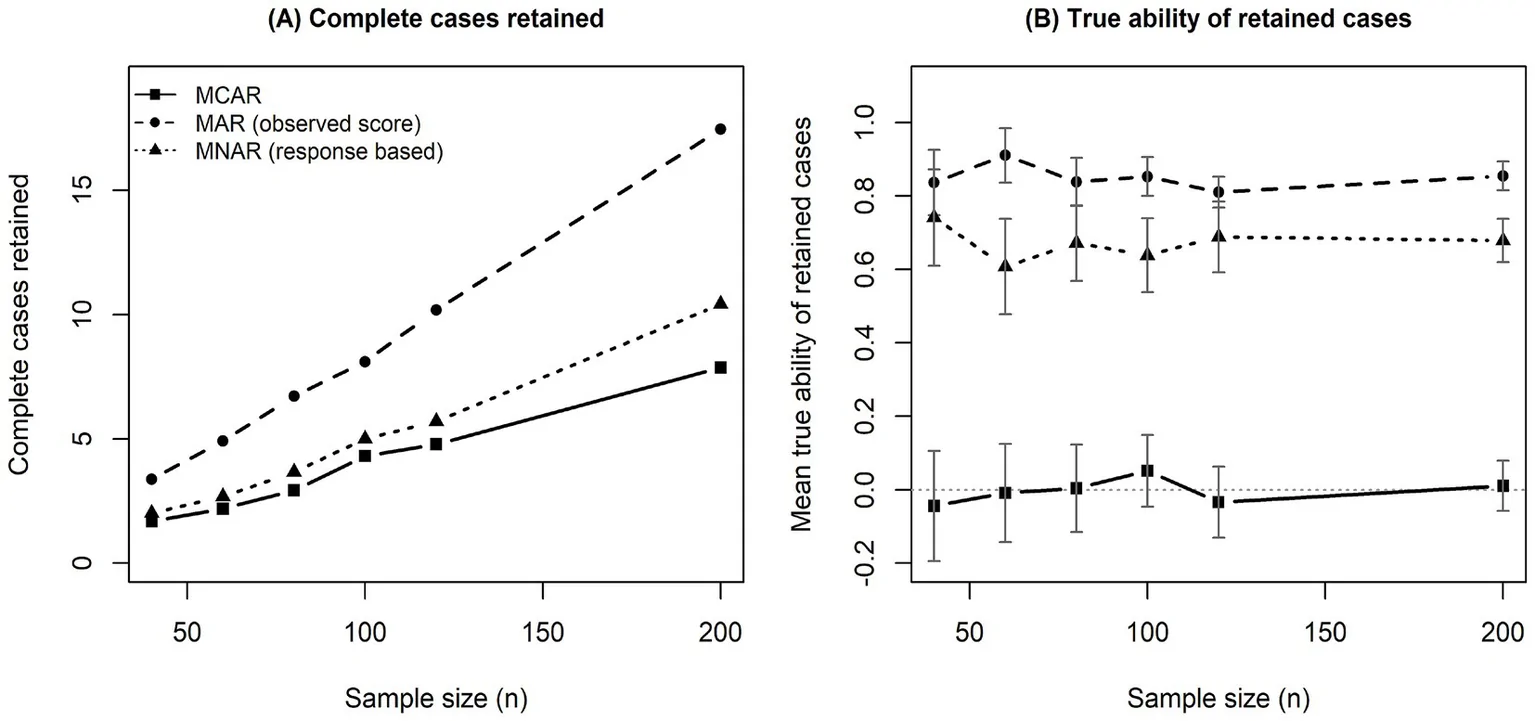
Listwise deletion at a 15% missingness rate. Panel **(A)** shows the mean number of complete cases retained. Panel **(B)** shows the mean true ability of the retained subsample, with bars giving 95% intervals.

The item response tree model coded each response into a participation node, observed for all examinees, and a correctness node, observed only for answered items, following Pohl et al. (2014) as operationalized by De Boeck and Partchev (2012). A participation factor was loaded on the first-node items and an ability factor on the second-node items; the two factors were free to correlate, and ability scores were taken from the second factor. The main estimator was Metropolis-Hastings Robbins-Monro with 1,000 cycles. A fourfold iteration budget and the expectation–maximization algorithm were run as diagnostics and do not enter any accuracy comparison. All estimation used the mirt package version 1.46.1 (Chalmers, 2012) and the mice package (Van Buuren and Groothuis-Oudshoorn, 2011) in R 4.5.2 (R Core Team, 2025), and item difficulties were extracted in the item response theory parameterization throughout.

### Correctly specified experiment

To separate small-sample effects from possible misspecification, an additional condition generated data directly from the two-factor tree model with known node-specific parameters and a known factor correlation of 0.30, following the approach of Alarcon et al. (2023), and fitted the same model to those data. The generating parameters were fixed in advance and verified against a large pilot sample before any small-sample estimation was attempted, so they could not be adjusted to improve convergence. The pilot confirmed a marginal omission rate of approximately 0.13.

### Evaluation

Two outcomes were separated throughout: the probability of obtaining an admissible solution and accuracy conditional on obtaining one. Recovery measures were computed within each replication and aggregated to the cell level as a separate step. Item-parameter recovery against the generating truth was evaluated for the three procedures based on the two-parameter logistic model; for the tree model, whose node parameters have no counterpart in a unidimensional generating model, item recovery was evaluated in the correctly specified experiment, and parameter stability was reported in all conditions. Paired comparisons were computed only on replications in which both procedures produced an admissible solution. No *post hoc* threshold was applied to these comparisons: the number of contributing pairs is reported in every cell, cells resting on fewer than 10 pairs are retained but labeled as highly uncertain, and comparisons with fewer than two pairs are not estimable. The paired difference is defined as the root mean square error of the first procedure minus that of the second, so that a positive value favors the second procedure, and this definition is used in every table, figure, and sentence. Confidence intervals are paired intervals against zero. Estimability proportions are reported separately from conditional accuracy so that the selection induced by conditioning on successful estimation remains visible.

## Results

### Estimation feasibility

Estimability, not accuracy, separated the procedures. Table 1 reports the proportion of replications yielding an admissible solution at a 15% missingness rate. The ordering is identical in every cell of the design: the complete-data benchmark is most often estimable, followed by direct estimation, then multiple imputation, then the tree model Figure 3.

**Table 1:** Proportion of replications yielding an admissible solution, 15% missingness.

*n*	Mechanism	Complete data	Direct	MI	IRTree
40	MCAR	0.46	0.23	0.07	0.00
60	MCAR	0.66	0.50	0.41	0.08
80	MCAR	0.96	0.87	0.76	0.33
100	MCAR	0.95	0.92	0.85	0.71
120	MCAR	1.00	0.98	0.93	0.83
200	MCAR	0.99	0.99	0.97	0.99
40	MAR	0.42	0.19	0.02	0.05
60	MAR	0.76	0.50	0.32	0.22
80	MAR	0.92	0.77	0.63	0.60
100	MAR	0.99	0.96	0.81	0.83
120	MAR	0.99	0.98	0.87	0.92
200	MAR	1.00	0.98	0.98	0.99
40	MNAR	0.42	0.22	0.03	0.00
60	MNAR	0.83	0.61	0.41	0.18
80	MNAR	0.92	0.80	0.69	0.37
100	MNAR	0.98	0.93	0.85	0.73
120	MNAR	0.99	0.98	0.90	0.86
200	MNAR	1.00	1.00	0.97	0.97

MCAR, missing completely at random; MAR, missing at random, generated from observed total-score groups; MNAR, non-ignorable, generated from the response. Complete data = benchmark fitted before missingness was imposed. Direct = two-parameter logistic model fitted to the incomplete matrix. MI, multiple imputation followed by calibration, counted as successful only when all five imputations yielded an admissible calibration. IRTree = two-factor item response tree, Metropolis-Hastings Robbins-Monro with 1,000 cycles. Based on 100 replications per cell.

**Figure 3:**
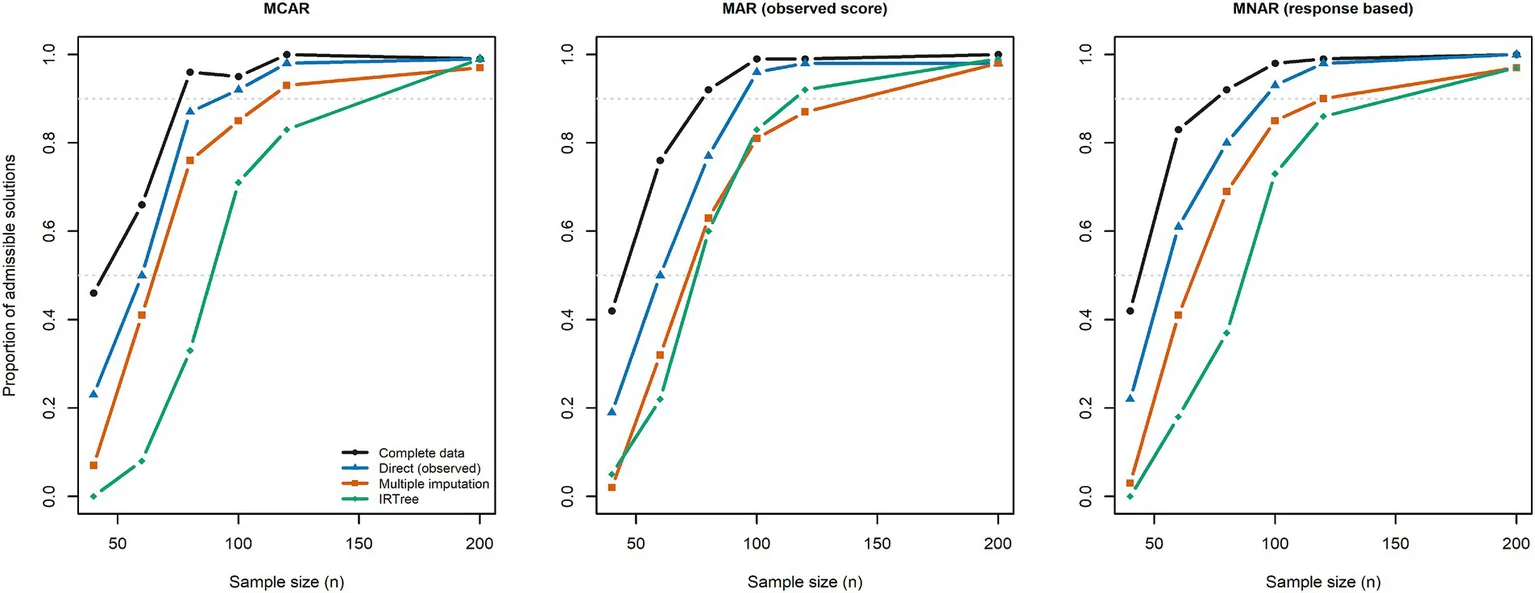
Proportion of replications yielding an admissible solution, by sample size, procedure, and mechanism.

At 40 examinees, the complete-data benchmark itself failed in more than half of the replications. This is the most consequential single result in the study, because it shows that the difficulty at this scale is not created by missing responses. It is a property of calibrating a two-parameter logistic model with 20 items and 40 examinees, and every missing-data procedure inherits it. The corresponding figures for the procedures that must work with incomplete data are lower still, and the tree model is admissible in at most 5% of replications.

The failures were not of a single kind. Across the whole tree-model design, 54.4% of fits were admissible, 37.6% failed to converge, 4.4% ended in sampler failure, 2.5% were rejected because an item in the observed matrix had no variance, and 1.0% failed because the participation indicator for some item had no variance, that is, because every examinee happened to answer that item. The last category is specific to the tree parameterization and is a structural rather than a numerical limitation: with 40 examinees and a 15% omission rate, the omission indicator for an individual item is not guaranteed to vary. Failures of every kind were concentrated at the smallest samples, and at 200 examinees, only 14 of 800 fits failed to converge and no other failure type occurred.

### Listwise deletion

Listwise deletion was not a usable option anywhere in the design. With 20 items and 15% independent missingness, the expected complete-case proportion is 0.85 raised to the twentieth power, which is approximately 0.039, and the simulation matched this expectation. Mean retained cases ranged from 1.7 at a sample size of 40 to 17.5 at 200. At a sample size of 40 under the completely random mechanism, 17% of replications retained no complete case at all.

More important than the loss of cases is the selectivity of what remains. Under the missing-at-random mechanism, the retained subsample had a mean true ability between 0.81 and 0.91 standard deviations above the population mean, and under the response-based mechanism, between 0.61 and 0.74, whereas under the completely random mechanism it was approximately zero. The survivors of listwise deletion are the higher-achieving students who omitted the fewest items, so the retained sample misrepresents the population the assessment is intended to evaluate. For this reason, listwise deletion was excluded from the accuracy comparison and is reported as a feasibility and selectivity result only.

### Item calibration

Conditional on an admissible solution, item calibration was poor at the smallest samples for every procedure, as Table 2 shows.

**Table 2:** Item calibration conditional on an admissible solution, averaged across the three main mechanisms.

Procedure	*n*	Admissible replications	Max |a|	RMSE a	RMSE b (median)
Complete data	40	130	5.77	1.29	0.64
Complete data	60	225	5.32	1.12	0.49
Complete data	80	280	4.00	0.76	0.39
Complete data	100	292	3.45	0.61	0.35
Complete data	120	298	3.05	0.51	0.29
Complete data	200	299	2.64	0.36	0.22
Direct	40	64	9.86	2.34	0.78
Direct	60	161	5.40	1.17	0.58
Direct	80	244	4.54	0.92	0.45
Direct	100	281	4.05	0.77	0.41
Direct	120	294	3.39	0.61	0.33
Direct	200	297	2.81	0.42	0.26
MI	40	12	4.15	0.96	1.12
MI	60	114	3.80	0.84	0.99
MI	80	208	3.59	0.72	0.55
MI	100	251	3.31	0.63	0.46
MI	120	270	3.06	0.54	0.35
MI	200	292	2.70	0.40	0.28
IRTree	40	5	10.07	n.a.	n.a.
IRTree	60	48	4.42	n.a.	n.a.
IRTree	80	130	4.12	n.a.	n.a.
IRTree	100	227	4.04	n.a.	n.a.
IRTree	120	261	3.68	n.a.	n.a.
IRTree	200	295	2.99	n.a.	n.a.

RMSE, root mean square error. Values are averaged across the three main mechanisms at a 15% missingness rate; mechanism-specific values are given in the Supplementary material. The generating ceiling for discrimination is approximately 2. Median root mean square error is reported for difficulty because the mean is dominated by occasional items whose discrimination approaches zero, which leaves that difficulty unidentified. For the tree model fitted to data generated from a unidimensional model, there is no generating counterpart for the node parameters, so item recovery is not defined; it is reported instead in the correctly specified experiment of Table 5.

With complete data at 40 examinees, the mean maximum absolute discrimination was 5.77 against a generating ceiling of about 2, and the root mean square error for discrimination was 1.29. By 200 examinees, these values had fallen to 2.64 and 0.36. The same pattern holds for every procedure, including the benchmark, so the behavior is a small-sample phenomenon rather than a property of any one method. Figure 4 shows the corresponding pattern for the tree model, separately by mechanism and by factor.

**Figure 4:**
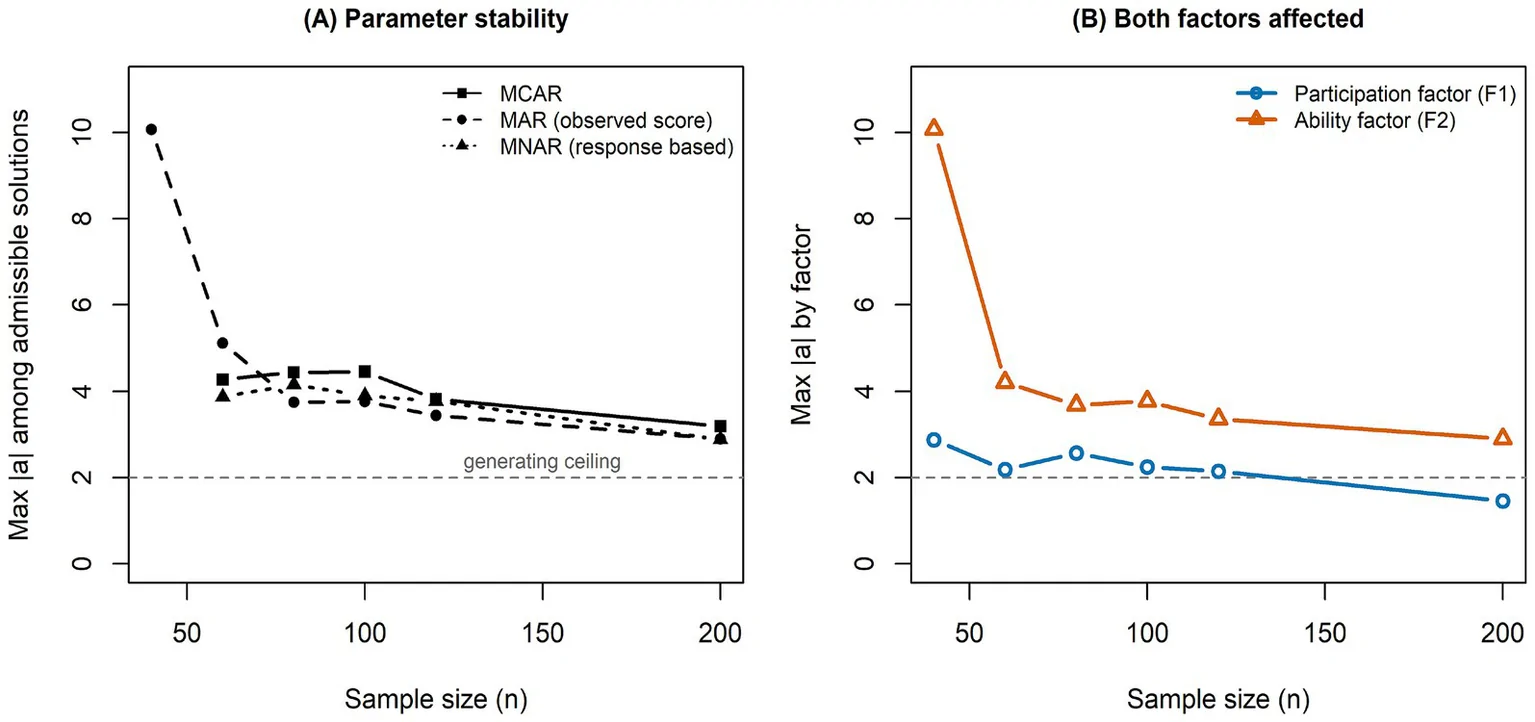
Stability of the item response tree model. Panel **(A)** shows the mean maximum absolute discrimination among admissible solutions against the generating ceiling. Panel **(B)** separates the participation and ability factors.

### Ability recovery

Once estimation succeeded, the procedures were close to one another, and substantial individual-level uncertainty remained throughout. Table 3 and Figure 5 reports the conditional results.

**Table 3:** Ability recovery conditional on an admissible solution, averaged across the three main mechanisms.

Procedure	*n*	Admissible replications	RMSE	Correlation with true ability
Complete data	40	130	0.446	0.908
Complete data	60	225	0.429	0.911
Complete data	80	280	0.415	0.914
Complete data	100	292	0.408	0.916
Complete data	120	298	0.406	0.919
Complete data	200	299	0.399	0.920
Direct	40	64	0.481	0.891
Direct	60	161	0.459	0.894
Direct	80	244	0.449	0.898
Direct	100	281	0.442	0.900
Direct	120	294	0.437	0.904
Direct	200	297	0.429	0.906
MI	40	12	0.500	0.878
MI	60	114	0.467	0.888
MI	80	208	0.457	0.894
MI	100	251	0.449	0.896
MI	120	270	0.446	0.900
MI	200	292	0.438	0.902
IRTree	40	5	0.504	0.871
IRTree	60	48	0.469	0.887
IRTree	80	130	0.458	0.890
IRTree	100	227	0.447	0.897
IRTree	120	261	0.443	0.901
IRTree	200	295	0.433	0.904

RMSE, root mean square error. Results are conditional on an admissible solution and are averaged across the three main mechanisms at a 15% missingness rate. The study did not prespecify a decision-specific adequacy threshold, so these values are reported without a verdict on sufficiency for any particular educational use. Estimability is reported separately in Table 1.

**Figure 5:**
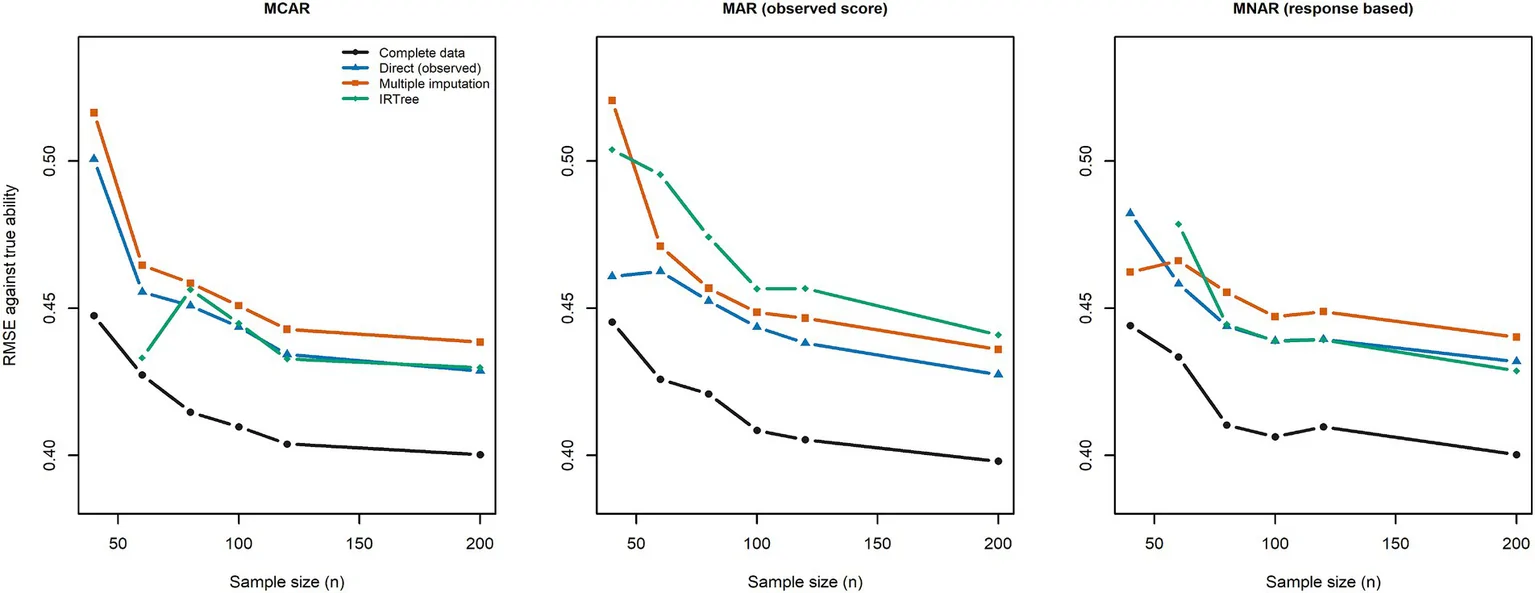
Root mean square error against true ability, conditional on an admissible solution, by sample size, procedure, and mechanism.

Root mean square error against true ability ranged from 0.399 to 0.446 for the complete-data benchmark, 0.429 to 0.481 for direct estimation, 0.438 to 0.500 for multiple imputation, and 0.433 to 0.504 for the tree model. The correlation between estimated and true ability lay between 0.87 and 0.92. The study did not prespecify a decision-specific adequacy threshold, but these values indicate that substantial individual-level uncertainty remained even when estimation succeeded.

The paired comparisons make the ordering explicit. The difference between direct estimation and the complete-data benchmark lay between 0.028 and 0.052 in every cell, with intervals excluding zero, which quantifies the additional loss attributable to missingness and its treatment as small and remarkably stable across sample sizes and mechanisms. Table 4 and Figure 6 reports the two comparisons of primary interest.

**Table 4:** Paired differences in root mean square error against true ability, with the number of contributing replications.

*n*	Mechanism	Comparison	Pairs	Difference	95% CI	Precision
40	MCAR	MI vs. Direct	6	0.024	[0.009, 0.040]	Highly uncertain
40	MCAR	IRTree vs. Direct	0	–	–	Not estimable
60	MCAR	MI vs. Direct	33	0.009	[0.002, 0.016]	
60	MCAR	IRTree vs. Direct	7	0.003	[−0.005, 0.010]	Highly uncertain
80	MCAR	MI vs. Direct	74	0.010	[0.007, 0.013]	
80	MCAR	IRTree vs. Direct	33	0.001	[−0.001, 0.003]	
100	MCAR	MI vs. Direct	82	0.010	[0.007, 0.012]	
100	MCAR	IRTree vs. Direct	70	0.003	[0.001, 0.005]	
120	MCAR	MI vs. Direct	93	0.010	[0.008, 0.012]	
120	MCAR	IRTree vs. Direct	83	0.002	[0.000, 0.003]	
200	MCAR	MI vs. Direct	97	0.009	[0.008, 0.010]	
200	MCAR	IRTree vs. Direct	99	0.001	[0.000, 0.002]	
40	MAR	MI vs. Direct	1	–	–	Not estimable
40	MAR	IRTree vs. Direct	3	0.045	[−0.036, 0.127]	Highly uncertain
60	MAR	MI vs. Direct	28	0.011	[0.002, 0.020]	
60	MAR	IRTree vs. Direct	18	0.035	[0.021, 0.049]	
80	MAR	MI vs. Direct	60	0.008	[0.004, 0.012]	
80	MAR	IRTree vs. Direct	58	0.023	[0.017, 0.029]	
100	MAR	MI vs. Direct	81	0.008	[0.005, 0.012]	
100	MAR	IRTree vs. Direct	83	0.015	[0.011, 0.019]	
120	MAR	MI vs. Direct	87	0.011	[0.009, 0.014]	
120	MAR	IRTree vs. Direct	92	0.020	[0.017, 0.024]	
200	MAR	MI vs. Direct	98	0.009	[0.007, 0.010]	
200	MAR	IRTree vs. Direct	98	0.013	[0.010, 0.015]	
40	MNAR	MI vs. Direct	2	0.037	[−0.316, 0.390]	Highly uncertain
40	MNAR	IRTree vs. Direct	0	–	–	Not estimable
60	MNAR	MI vs. Direct	37	0.013	[0.007, 0.020]	
60	MNAR	IRTree vs. Direct	18	0.023	[0.011, 0.035]	
80	MNAR	MI vs. Direct	64	0.015	[0.012, 0.019]	
80	MNAR	IRTree vs. Direct	37	0.006	[0.001, 0.011]	
100	MNAR	MI vs. Direct	85	0.011	[0.008, 0.014]	
100	MNAR	IRTree vs. Direct	72	0.004	[0.000, 0.007]	
120	MNAR	MI vs. Direct	90	0.010	[0.008, 0.013]	
120	MNAR	IRTree vs. Direct	86	0.002	[−0.000, 0.005]	
200	MNAR	MI vs. Direct	97	0.009	[0.008, 0.010]	
200	MNAR	IRTree vs. Direct	97	−0.003	[−0.004, −0.001]	

RMSE, root mean square error; CI, confidence interval. The difference is the root mean square error of the first procedure minus that of the second, so a positive value favors the second. Comparisons use only replications in which both procedures produced an admissible solution. No post hoc threshold was applied; the number of contributing pairs is reported for every cell, and cells resting on fewer than 10 pairs are labeled highly uncertain. The comparison of direct estimation against the complete-data benchmark and all remaining comparisons are given in Supplementary Table S1.

**Figure 6:**
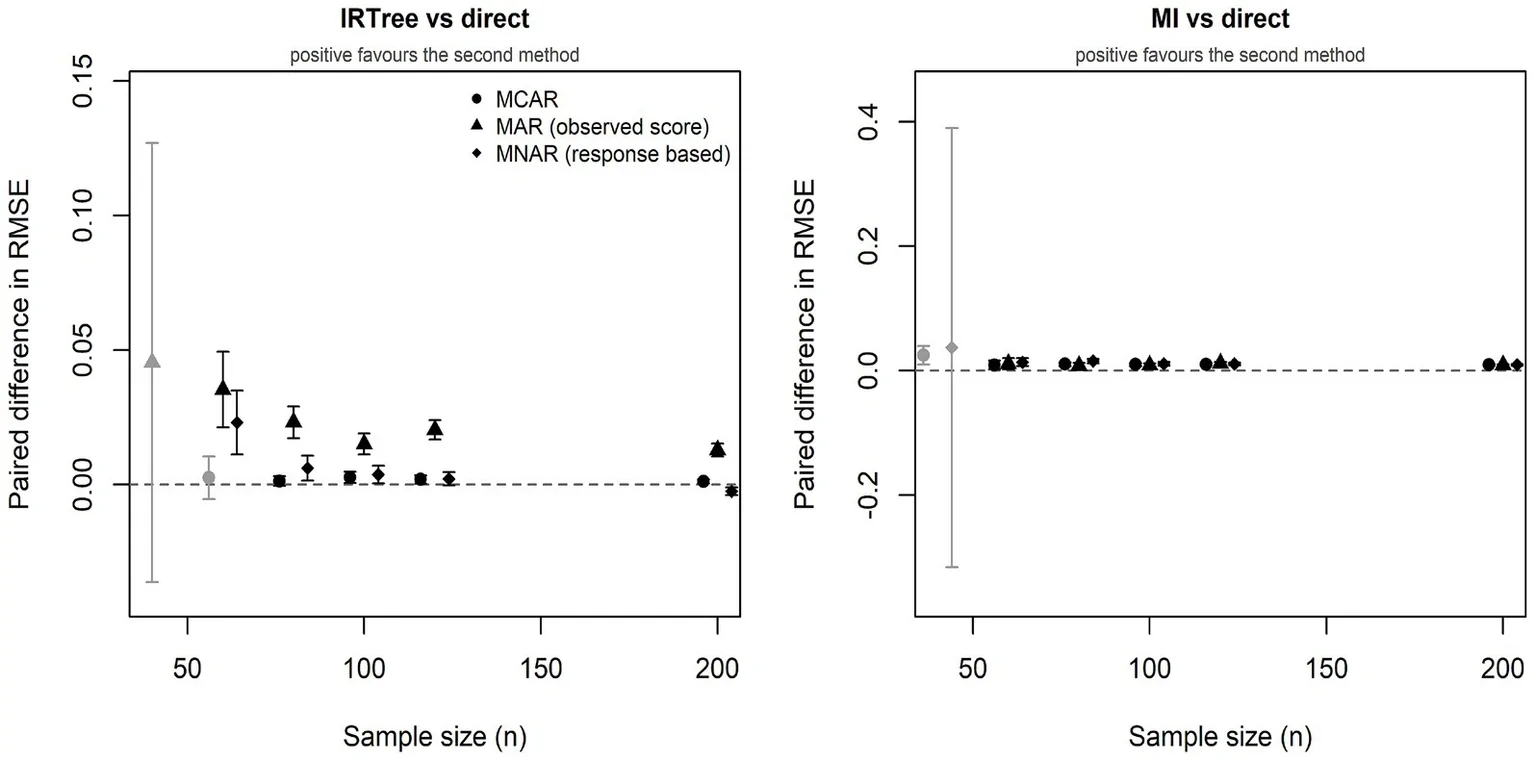
Paired differences in root mean square error with 95% intervals. Positive values favor the second procedure named. Comparisons with fewer than two paired replications are not estimable; those based on two to nine pairs are retained but labeled as highly uncertain.

Wherever the comparison rested on more than a handful of replications, multiple imputation was less accurate than direct estimation by between 0.008 and 0.015, with intervals excluding zero. Under the investigated conditions, therefore, direct estimation outperformed the implemented predictive mean matching procedure, a result in the same direction as the large-sample finding of Xiao and Bulut (2020).

The comparison between multiple imputation and direct estimation deserves one qualification, because the two are not symmetric procedures. A single-fit and a five-fit procedure differ in how easily they can fail. When the per-imputation admissibility rate is compared with the single fit of the direct analysis, the ordering reverses: at 40 examinees, an individual imputed data set yielded an admissible calibration in 0.33 to 0.37 of cases, against 0.19 to 0.23 for the incomplete matrix. Imputation makes each calibration easier, because the matrix it produces is complete, while making the procedure as a whole more fragile, because all five calibrations must succeed. Under a relaxed rule requiring three of five, the replication-level success rate at 40 examinees rises from between 0.02 and 0.08 to between 0.28 and 0.34. Both quantities are reported in the Supplementary material so that readers can apply whichever rule matches their practice.

The tree model, where it could be estimated, offered no dependable advantage. Against direct estimation, the paired difference was between 0.001 and 0.003 under the completely random mechanism, between 0.013 and 0.035 in favor of direct estimation under the missing at random mechanism, and in favor of direct estimation under the response-based mechanism at all sample sizes except 200, where the tree model was better by 0.003. At 40 examinees, the comparison rests on at most three replications and is not informative. The single cell in which the model designed to exploit informative omission outperformed a conventional analysis is therefore the largest sample under the mechanism it was designed for, and the margin there is small relative to a root mean square error of about 0.43.

### The breakdown is not explained by misspecification

A natural objection to the preceding results is that the tree model was fitted to data generated from a unidimensional model, so its failure might reflect the mismatch between the fitted missing-propensity model and the process that generated missingness rather than sample size as such. This objection is well founded in general, since Pohl and Becker (2020) showed that performance depends strongly on that correspondence. It was tested directly (see Table 5).

**Table 5:** Estimability of the two-factor tree model under misspecification and under correct specification.

*n*	Data from 2PL	Data from IRTree	Max |a| when admissible	Estimated factor correlation
40	0.02	0.00	Not estimable	Not estimable
60	0.16	0.02	4.63	0.36
80	0.43	0.22	4.45	0.30
100	0.76	0.54	3.93	0.29
120	0.87	0.73	4.08	0.33
200	0.98	0.98	3.38	0.32

2PL, two-parameter logistic model; IRTree, item response tree. The second column averages the three mechanisms of the main design. In the correctly specified condition, the data are generated from the same two-factor tree model that is then fitted, with a true factor correlation of 0.30. Based on 100 replications per cell.

The breakdown persists under correct specification. When the data are generated from the two-factor tree model itself, with known node parameters fixed in advance, an admissible solution is obtained in 0.00 of replications at 40 examinees, 0.02 at 60, 0.22 at 80, 0.54 at 100, 0.73 at 120, and 0.98 at 200 (Figure 7). Where estimation succeeds, the estimated factor correlation lies between 0.29 and 0.36 against a true value of 0.30; however, the smallest-sample summaries rest on very few admissible replications and are therefore interpreted cautiously. The experiment nevertheless rules out misspecification as the sole explanation for the failures and is consistent with the interpretation that the freely estimated two-factor structure demands more information than these sample sizes provide.

**Figure 7:**
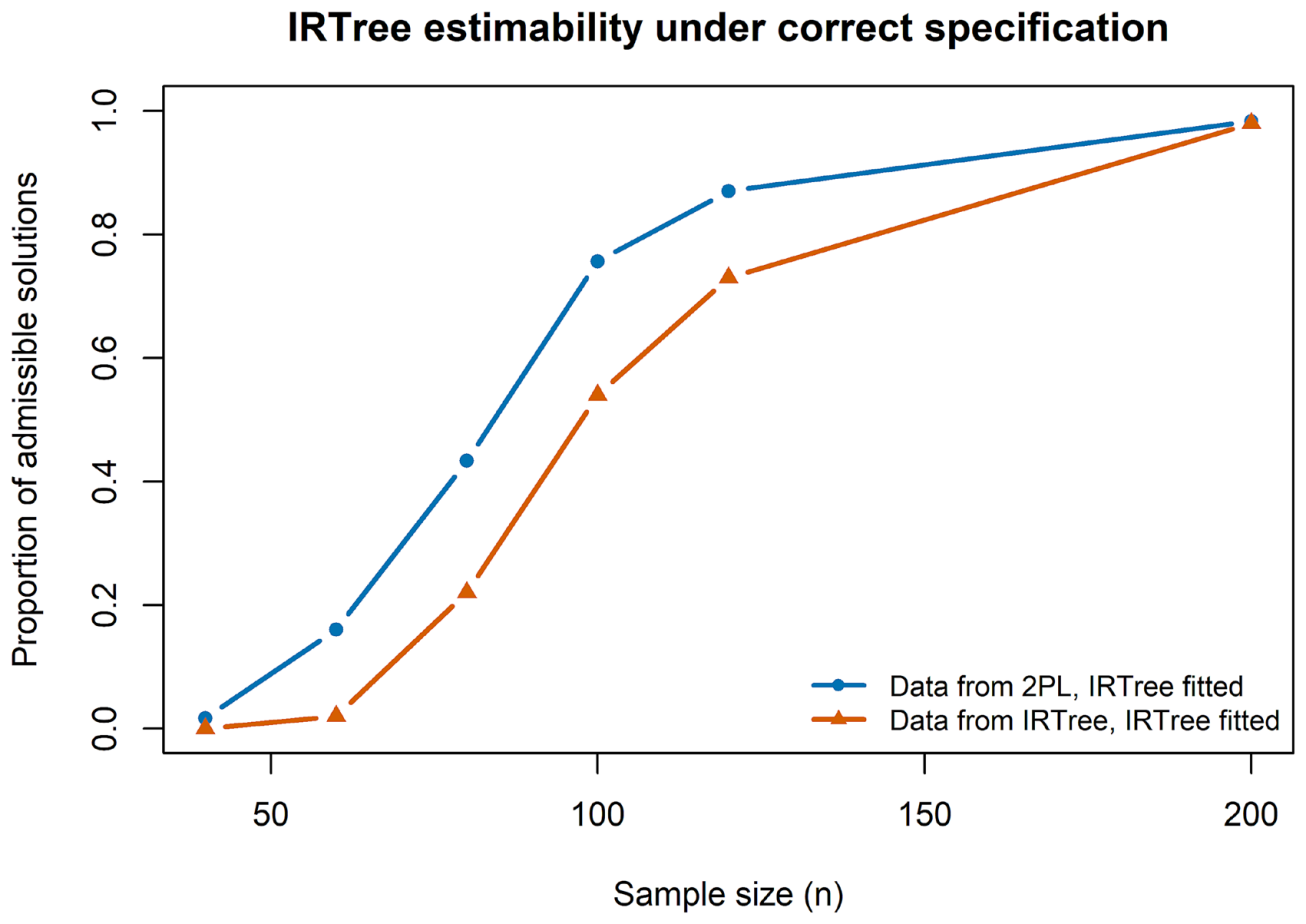
Estimability of the two-factor tree model when the data are generated from a unidimensional model and when they are generated from the tree model itself.

A second diagnostic addresses the same question from another direction. If the degeneracy arose from sparse information about omission, it should be concentrated in the participation factor. It is not. At the smallest samples, the maximum discrimination is inflated on both factors, and under the missing at random mechanism the ability factor is, if anything, the more affected of the two.

### A convergence indicator alone is not sufficient evidence

The estimator diagnostics produced a result with direct implications for practice. Table 6 and Figure 8 reports the three estimation settings at a sample size of 40.

**Table 6:** Estimator diagnostics at a sample size of 40, across the three main mechanisms.

Estimation setting	Admissible	Mean max |a|	95th percentile max |a|
MH-RM, 1,000 cycles	0.00 to 0.05	22.5 to 25.2	37.1 to 45.8
MH-RM, 4,000 cycles	0.05 to 0.20	22.6 to 26.4	40.5 to 48.5
EM, 2,000 cycles	0.73 to 0.82	19.0 to 20.0	29.0 to 34.1

MH-RM, Metropolis-Hastings Robbins-Monro; EM, expectation–maximization. Reported as a diagnostic. These settings do not enter any accuracy comparison. The generating ceiling for discrimination is approximately 2. Based on 100 replications per cell in each of three mechanisms.

**Figure 8:**
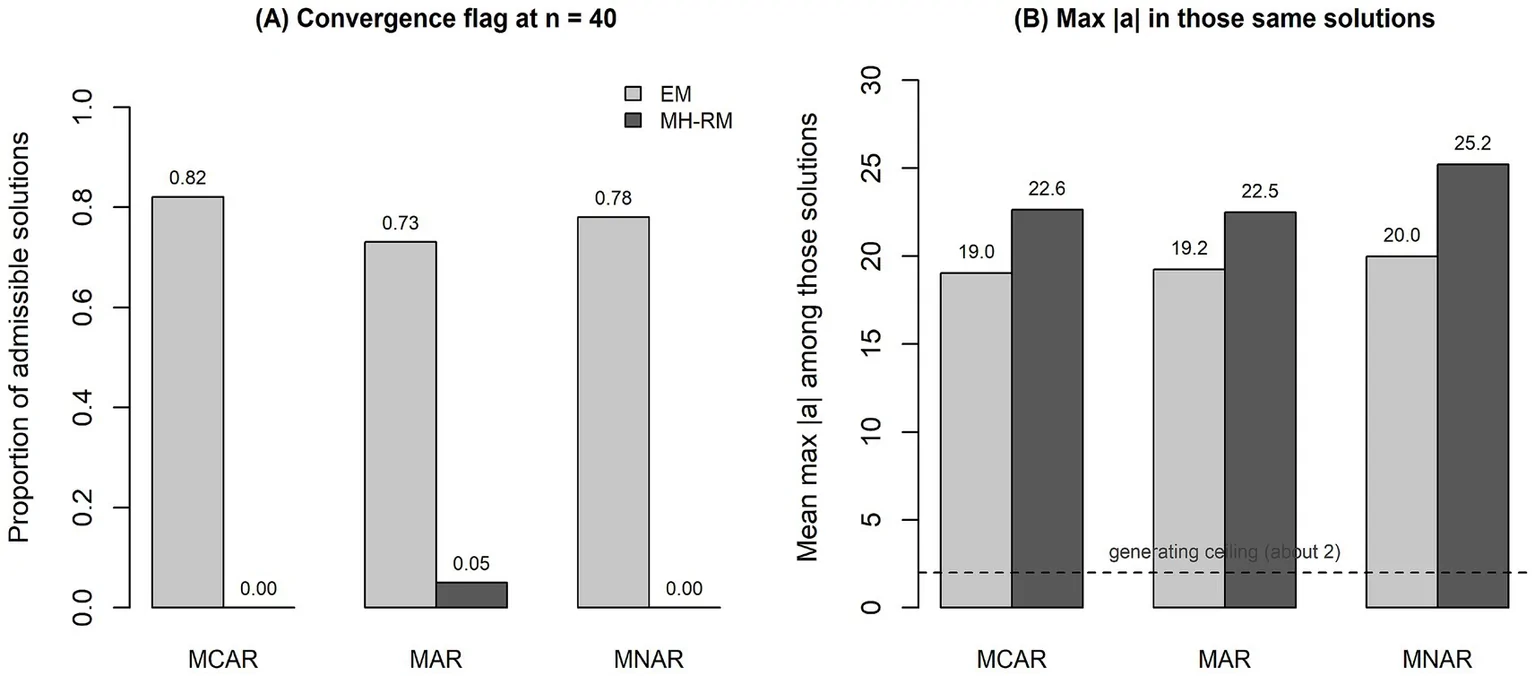
Estimator diagnostics at a sample size of 40. Panel **(A)** shows the proportion of admissible solutions. Panel **(B)** shows the mean maximum absolute discrimination in those same solutions, against the generating ceiling.

Quadrupling the iteration budget did not restore estimation; it raised the admissible proportion modestly while pushing the discriminations further into the degenerate region. The expectation–maximization algorithm presents the more instructive case. It reported an admissible solution in 0.73 to 0.82 of replications, an order of magnitude more often than the sampling-based estimator, yet those same solutions carried a mean maximum discrimination near 19 and a 95th percentile between 29 and 34. Such a result shows that a convergence flag alone does not establish stable parameter estimation. This is the strongest practical argument in the study for reporting convergence and parameter behavior together, and it also illustrates why admissibility is treated as an operational completion criterion rather than a verdict on parameter quality.

### Sensitivity conditions

At a 25% missingness rate, the ordering of the procedures is unchanged, and every proportion is lower. At 40 examinees, direct estimation yielded an admissible solution in 0.08 to 0.11 of replications, multiple imputation in 0.01 to 0.02, and the tree model in 0.00 to 0.03. At 120 examinees, the corresponding figures were 0.86 to 0.94, 0.71 to 0.77, and 0.76 to 0.82, and at 200 examinees, 0.95 to 0.99, 0.93 to 0.95, and 0.97 to 0.99. Under the latent-ability-dependent non-ignorable mechanism retained as a sensitivity condition, the complete-data benchmark yielded an admissible solution in 0.47 of replications at 40 examinees and 0.84 at 60, and the ordering of the procedures again matched the main design.

## Discussion

The central result of this study is that at classroom scale, the choice of a missing-data strategy is not settled by accuracy. Once a procedure produced an admissible solution, the procedures differed by only a few hundredths of a theta standard deviation in root mean square error. What separated them much more strongly was whether they could be estimated at all. A framework that ranks procedures by root mean square error alone therefore emphasizes the dimension along which they differ least.

This reframing has a direct consequence for the recommendation that motivated the study. The advantage reported for tree-based models at samples of 400 and above (Soğuksu and Demir, 2025) does not extend downward. The freely estimated two-factor model with 20 items yields an admissible solution in at most 0.05 of replications at 40 examinees and does not reach 0.90 in any mechanism until 120 examinees or beyond. The conclusion by Soğuksu and Demir (2025) that sample size was not a substantial factor may hold within the range they investigated but should not be extrapolated to samples an order of magnitude smaller, where sample size governs not accuracy but estimability. The correctly specified experiment and the factor-specific diagnostics indicate that the observed instability cannot be explained solely by generating-model misspecification or by a problem confined to the participation factor.

We do not claim a general threshold below which tree models are intrinsically under-identified. What the evidence supports is narrower and more useful: the freely estimated two-factor specification with 20 items becomes severely unstable below approximately 100 examinees under the conditions investigated here, and misspecification can be ruled out as the sole explanation. A constrained parameterization, for instance, one fixing node discriminations, might be estimable with smaller samples, but it would be a different model and would have to be evaluated on its own terms. Longer tests supply more information per examinee and might shift the boundary.

The second finding concerns imputation. Under the investigated conditions, the implemented predictive mean matching procedure was less often estimable and slightly less accurate than fitting the measurement model directly to the incomplete matrix. The mechanism behind this is worth stating clearly because it is easy to misread. Imputation does not fail at the imputation step. It makes each calibration easier, since the matrix it produces is complete. What it does is insert an additional layer that must succeed five times over, and it discards the information that a response was omitted rather than incorrect. At classroom scale, the layer costs more than it returns. This reproduces at small scale what Xiao and Bulut (2020) reported at large scale, and it is consistent with the discussion of ignoring omitted responses in Pohl et al. (2014). Whether other imputation implementations behave differently was not examined here.

The third finding is the most uncomfortable and most important for practice. The complete-data benchmark failed in more than half of replications at 40 examinees, with discriminations inflated well above their generating ceiling. Şahin and Anıl (2017) reported that 500 respondents were needed for their item-parameter recovery criteria with a 20-item two-parameter logistic test, and although their figure is conditional on their setting, the direction of the present results is the same. No missing-data strategy can repair a calibration that the sample cannot support. An evaluator working with a single class who fits an item response model and inspects only the resulting ability estimates will see plausible numbers, because expected a posteriori scoring with a standard normal prior shrinks person estimates toward the mean and conceals the failure at the level of scores. The failure is visible only in convergence behavior and item parameters. That is why reporting convergence rates and parameter stability should be standard practice whenever these models are applied to small assessments, and why the estimator comparison reported above matters: an estimator that reports convergence while returning discriminations near 19 will mislead anyone who trusts the flag alone.

For listwise deletion, the conclusion is unambiguous within this design. It is not merely inefficient at this scale; it retains too few cases to analyze, and where it retains more cases, it retains a biased subset of higher-achieving students. Its use as a default for classroom assessment data with moderate missingness is difficult to justify.

### Implications for practice

Three suggestions follow, each stated for the conditions investigated here. Do not use listwise deletion when it leaves only a small and selective complete-case subset. Prefer fitting the measurement model to the responses that were observed over adding the implemented imputation layer, because the layer showed no supported accuracy benefit in this design. For the freely estimated 20-item two-factor tree model, admissibility did not become consistently high until the upper end of the examined sample-size range; applied users should therefore verify convergence and parameter stability rather than apply a universal numerical cutoff. Above all, report estimation behavior and not only scores. A convergence rate, a maximum discrimination, and the number of analyses that failed provide essential information about whether the measurement model was adequately estimated.

### Limitations

The study uses one test length of 20 items, one set of item-parameter distributions, one standard normal ability distribution, and one imputation implementation, namely predictive mean matching with five imputations and ten iterations followed by calibration and averaging. Missingness rates of 15 and 25% were examined, with the latter evaluated only at three sample sizes. The response-score-based missing-at-random condition is an operationalization based on a total score computed before masking and should not be interpreted as establishing how missingness arises in real classroom data. The conclusions therefore apply to the implemented procedures and generating conditions rather than to all missing-data methods or IRTree specifications. Test length and sample size jointly determine the amount of information available for calibration, so longer tests may shift the sample-size region in which estimation becomes stable. The tree model was evaluated in a freely estimated 20-item two-factor form; constrained variants and the mixture and dynamic extensions described by Alagöz and Meiser (2024) and Merhof and Meiser (2023) were not examined. The correctly specified IRTree experiment likewise represents one generating parameterization and one factor correlation. Finally, accuracy results are conditional on successful estimation, and at the smallest samples, this conditioning may retain the more tractable replications. Estimability rates are therefore reported separately so that readers can see how much selection precedes the conditional accuracy summaries.

## Conclusion

For classroom-scale student assessments, the strategy that performs best at large samples is not necessarily the strategy to adopt by default. The freely estimated 20-item two-factor item response tree model showed severe estimation instability throughout the smallest sample-size region, and that pattern persisted when the data were generated from the same tree model with known parameters. The implemented imputation procedure was more fragile than fitting the measurement model directly to the incomplete matrix and showed no supported accuracy advantage in the adequately informed paired comparisons—listwise deletion retained too few cases and, under informative mechanisms, a selective subset. The most consequential result is that at 40 examinees, calibration failed in more than half of replications even with complete data, so no treatment of missingness can remove the underlying small-sample calibration problem. At this scale, the useful first question is whether the model can be estimated, and evaluators should report convergence and parameter stability alongside any ability estimates they present.

## Data Availability

The complete R simulation code, the frozen analysis protocol and its timestamped record, the technical verification report, the data-generation quality-control output, the replication-level raw logs, and the aggregation, figure and table scripts are provided as Supplementary material. Every value reported in this article can be regenerated from the raw logs without re-running the simulation, and any individual condition can be reproduced on its own.
